# Author Correction: REST regulates the cell cycle for cardiac development and regeneration

**DOI:** 10.1038/s41467-017-02617-7

**Published:** 2018-01-12

**Authors:** Donghong Zhang, Yidong Wang, Pengfei Lu, Ping Wang, Xinchun Yuan, Jianyun Yan, Chenleng Cai, Ching-Pin Chang, Deyou Zheng, Bingruo Wu, Bin Zhou

**Affiliations:** 1 Department of Genetics, Albert Einstein College of Medicine, Bronx, NY 10461 USA; 2 Department of Medical Ultrasound, The First Affiliated Hospital of Nanchang University, Nanchang, 330006 China; 3 Department of Developmental and Regenerative Biology, The Black Family Stem Cell Institute, and The Mindich Child Health and Development Institute, Icahn School of Medicine at Mount Sinai, New York, NY 10029 USA; 4 Department of Medicine, Indian University School of Medicine, Indianapolis, IN 46202 USA; 5 Departments of Genetics, Neurology and Neuroscience, Albert Einstein College of Medicine, Bronx, NY 10461 USA; 6 Departments of Genetics, Pediatrics, and Medicine (Cardiology), The Wilf Cardiovascular Research Institute, The Institute for Aging Research, Albert Einstein College of Medicine, Bronx, NY 10461 USA; 7 Department of Cardiology, The First Affiliated Hospital and State Key Laboratory of Reproductive Medicine of Nanjing Medical University, Nanjing, Jiangsu 210029 China

**Keywords:** Cell growth, Cell proliferation, Cardiac regeneration, Heart development

Correction to: *Nature Communications* 10.1038/s41467-017-02210-y, published online 07 December 2017.

The original version of this article contained an error in the spelling of the author Jianyun Yan, which was incorrectly given as Jiangyun Yan. This has now been corrected in both the PDF and HTML versions of the article.

